# Social Behavior and Neurogenesis

**DOI:** 10.3390/ijms27052471

**Published:** 2026-03-07

**Authors:** Alejandro Tapia-De-Jesús, Mario Humberto Buenrostro-Jáuregui, Jesús Armando Mata-Luévanos

**Affiliations:** 1 Department of Health, Universidad Iberoamericana, Mexico City 01219, Mexico; 2 Laboratory of Neuroscience, Department of Psychology, Universidad Iberoamericana, Mexico City 01219, Mexico; mario.buenrostro@ibero.mx; 3 Laboratory of Integration in Motivation, Emotion and Neuroscience, Facultad de Medicina y Ciencias Biomédicas, Universidad Autónoma de Chihuahua, Chihuahua 31125, Mexico

**Keywords:** adult neurogenesis, social behavior, neuroendocrine modulation

## Abstract

Adult neurogenesis is a regulated form of brain plasticity shaped by interactions between hormonal systems and environmental context. Social experience has been identified as an important modulator of neuronal proliferation, differentiation, and survival across the lifespan, although effects vary across species, developmental stages, and experimental paradigms. This review synthesizes evidence indicating that diverse social behaviors—including isolation, social hierarchy, parenting, sexual interaction, social buffering, and social learning—engage neuroendocrine, neurochemical, and stress-related pathways that are associated with modulation of hippocampal and olfactory neurogenesis. Affiliative and reproductive contexts have been linked in multiple models to enhanced neurogenic indices via gonadal hormones, oxytocinergic and vasopressinergic signaling, and neurotrophic mechanisms, whereas chronic isolation or social defeat has frequently been associated with reduced neurogenic markers, particularly within stress-sensitive regions of the ventral dentate gyrus. Sex differences further shape these patterns, reflecting both biological regulation and uneven sampling across paradigms. Comparative findings in prairie voles, eusocial mole-rats, nonhuman primates, songbirds, and teleost fish indicate that social organization can be accompanied by either increased or constrained neurogenic activity, depending on ecological pressures and life-history strategies. Collectively, the available evidence suggests that adult neurogenesis represents a context-dependent plastic process embedded within vertebrate social systems, while underscoring the need for integrative and evidence-graded interpretations.

## 1. Introduction

Neurogenesis is a continuous process in the brain that remains highly sensitive to a wide range of internal and external factors, making it critical for mammalian maturation, development, adaptation, and evolution [1,2]. The neurogenic process comprises multiple stages that are modulated by shared neurochemical and hormonal systems [3]. Endogenous factors include hormonal and neurochemical signals that promote the proliferation, growth, survival, and functional integration of new neurons, whereas exogenous factors encompass environmental elements—both stressful and appetitive—that can either trigger or inhibit neurogenic mechanisms [4,5].

A wide body of research has examined key external modulators of neurogenesis. Physical activity, particularly cardiovascular exercise, promotes the generation of new neurons through neurotrophic pathways [6,7,8] and enhances memory and learning functions [9]. Environmental enrichment is likewise associated with increased neuronal density in the dentate gyrus of the hippocampus [4,10]. More recently, social experience has emerged as a potent regulator of neurogenesis, influencing each stage of the process [11,12]. A deeper understanding of these pathways offers opportunities to develop novel stimulation- and treatment-based strategies for multiple human conditions.

Comprehensive frameworks of adult neurogenesis have been previously described [13]. Building on this foundation, the present review focuses specifically on how social interaction shapes neurogenic processes across different stages of life.

Although animal models provide controlled and mechanistically tractable approaches to studying social behavior, it is important to recognize their conceptual scope. Comparative neurobiological research indicates that across vertebrates, social behaviors such as reproduction, aggression, parental care, dominance interactions, and affiliation are regulated by conserved neural circuits, including the social decision-making network [14]. These paradigms capture evolutionarily conserved mechanisms underlying social interaction and motivational regulation.

However, human social phenomena are additionally shaped by language, symbolic representation, cultural transmission, and meta-cognitive processes that enable explicit mentalizing, reflective communication, and reputation management [15]. While these higher-order processes build upon conserved neurobiological substrates, they extend beyond the operational domains typically modeled in non-human species.

Accordingly, translational interpretations should be made with caution. The present review therefore emphasizes conserved neuroendocrine and circuit-level mechanisms through which social experience influences adult neurogenesis, while acknowledging that the full sociocultural complexity of human social life cannot be fully recapitulated in animal paradigms.

Review approach. We conducted a targeted literature search in PubMed and Scopus (from database inception to 2026) using combinations of terms including “adult neurogenesis”, “hippocampus”, “dentate gyrus”, “social isolation”, “social defeat”, “dominance”, “social buffering”, “parenting”, “mating”, “oxytocin”, and “glucocorticoids”. Priority was given to studies with direct neurogenesis readouts (e.g., BrdU, Ki67, DCX; proliferation/survival/maturation; DG/SVZ; dorsal/ventral DG when available), followed by mechanistic studies that explicitly tested candidate pathways relevant to neurogenic regulation. Behavioral-only studies were used to contextualize social phenotypes but were explicitly labeled as indirect. When findings were mixed, we summarized moderators (species/strain, sex, developmental timing, manipulation intensity/contact modality, and readout type) and used graded language to reflect evidence strength.

Throughout this review, “adult neurogenesis” is not treated as a unitary construct. When possible, we specify the component assessed (proliferation, survival, maturation/integration), the anatomical region (dentate gyrus vs. subventricular zone), and the methodological marker used (e.g., BrdU, Ki67, DCX), as different components may show divergent responses under similar social conditions.

## 2. Socialization

Social interaction is associated with numerous neuroprotective, cognitive, and mood-related benefits, particularly by stimulating and enhancing cognitive functions such as learning and memory [16]. It also plays a key role in reducing the impact of stress and decreasing anxiety- and depression-like behaviors [17]. This review synthesizes evidence on the relationship between social behavior and neurogenesis, identifying different forms of social behavior and their underlying mechanisms—ranging from neurotrophic factors to the hormonal pathways governed by the hypothalamic–pituitary–gonadal and hypothalamic–pituitary–adrenal axes.

A recent systematic review of adult neurogenesis and social behavior in rodents synthesized evidence indicating that social living conditions can bidirectionally modulate dentate gyrus neurogenic indices. Across included paradigms, social isolation and repeated social defeat were generally associated with reductions in neurogenesis-related markers, whereas social dominance and social enrichment were more often linked to increases, although results vary across models and readouts [11]. The same review also summarizes studies in which experimental reduction in adult neurogenesis (e.g., via irradiation or antimitotic approaches) is associated with impairments in social recognition and social memory, reinforcing the need to distinguish direct neurogenesis readouts from behavioral-only endpoints when interpreting social–neurogenic coupling [11].

Several elements contribute to social behavior, along with their associated mechanisms and manifestations, including social hierarchy, sexual interaction, parenting, and living in groups or in isolation. All of these domains have been examined in relation to the generation, survival, and functional integration of neurons. However, interactions with other variables—such as age, sex, and species—produce heterogeneous findings, underscoring the need for studies that systematically incorporate these moderating factors.

Social behavior is broadly understood to comprise both reinforcing and aversive components, although most behaviors involve a mixture of the two. For instance, receiving aggression exemplifies an aversive social experience, whereas mating typically represents a highly reinforcing one. Social status, in turn, reflects a combination of both motivational components.

## 3. Isolation

In most wildlife species, living in a social group provides several advantages, including improved access to territory, greater protection, enhanced performance, and more reliable food sources—factors that collectively contribute to well-being and survival [18]. To avoid misinterpretations of the effects of social behavior, it is essential to identify both the social role of individuals and the contextual characteristics of their environment, as isolation can be stressful in one situation yet relatively neutral—or even rewarding—in another [19,20].

Here, “social isolation” denotes physical housing in the absence of conspecific contact (visual, tactile, and/or olfactory), typically implemented chronically during adolescence or adulthood, and should be distinguished from social defeat or dominance-related stress paradigms.

A systematic review and meta-analysis focusing on the oxytocin system under social isolation shows that isolation is associated with measurable alterations in oxytocin biology, but that effect direction and magnitude vary across species/strains, tissues assessed (central vs. peripheral), and features of the isolation paradigm (duration, timing, and social context) [21]. This synthesis therefore supports oxytocin signaling as a plausible mechanistic conduit linking isolation to downstream brain plasticity, while also emphasizing substantive heterogeneity that limits one-direction generalization. In line with this evidence tiering, we treat isolation–oxytocin links as mechanistic-level evidence unless adult neurogenesis markers (e.g., BrdU/Ki67/DCX with region and component specification) are measured in parallel.

Social isolation has been consistently associated with measurable changes in brain function and plasticity, including alterations in synaptic transmission and reductions in neurogenesis. [22,23,24]. However, these outcomes depend strongly on developmental stage. Because neurogenesis varies across the lifespan—adolescents generate and integrate new neurons at substantially higher rates than adults [25]—isolation during early life versus adulthood leads to different consequences.

**Figure 1:**
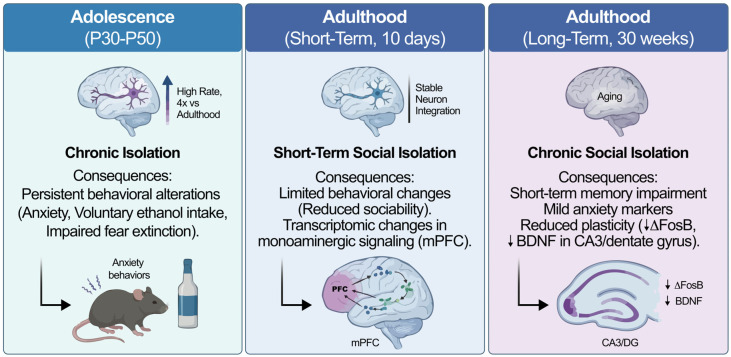
Age-dependent effects of social isolation on adult neurogenesis and behavior. Conceptual overview of age- and duration-dependent effects of social isolation on behavior and hippocampal plasticity. DG, dentate gyrus; CA3, cornu ammonis 3; mPFC, medial prefrontal cortex; BDNF, brain-derived neurotrophic factor.

Importantly, the neurogenic and behavioral impact of social isolation is strongly modulated by developmental stage and duration of exposure. Adolescence represents a particularly sensitive window, during which chronic isolation has been associated with persistent behavioral alterations and long-lasting vulnerability. In contrast, short-term isolation in adulthood produces relatively limited behavioral effects, whereas prolonged isolation has been linked to measurable impairments in hippocampal plasticity and cognition. These age-dependent patterns underscore that isolation does not exert uniform effects across the lifespan, but instead interacts with baseline neurogenic capacity and circuit maturation (Figure 1).

In addition to its effects on hippocampal plasticity, peripubertal social isolation has been associated with long-term alterations in reproductive neuroendocrine regulation and sexually motivated behavior. Experimental models in male rats isolated between postnatal days 25–50 show reduced adult sexual motivation, reflected in increased mount and intromission latencies, together with decreased serum testosterone levels [26,27]. Importantly, these endocrine alterations are accompanied by functional changes in prefrontal–amygdalar circuits: control males display a context-dependent association between testosterone levels and theta-band activity in the medial prefrontal cortex and basolateral amygdala, whereas this coupling is absent following peripubertal isolation [27]. These findings suggest that adolescent social deprivation may induce durable reorganization of neuroendocrine–limbic interactions, extending beyond hippocampal neurogenesis to circuits involved in motivation and reward processing.

Ibi et al. [22] conducted an experiment in which 3-week-old male mice were isolated for 4 weeks. When BrdU (bromodeoxyuridine) was administered at the end of the fourth week, no significant differences in cell proliferation were found between isolated and control groups. However, when BrdU was administered at the beginning of the fourth week, isolated mice showed significantly lower concentrations of BrdU-labeled cells in the dentate gyrus, indicating that isolation impaired cell differentiation and survival but not proliferation. Isolation is associated with different effects depending on natural fluctuations in gonadal hormones, sex, and specific features of the isolation paradigm, including onset and duration [28,29]. McCormick et al. [30] reported decreased cell proliferation and survival in the dentate gyrus during breeding [31] and adolescence [32]. These reductions, however, could be reversed by re-exposure to social housing during these developmental windows, consistent with heightened neuroplasticity during early life. In contrast, male mice isolated after weaning exhibited no changes in dentate gyrus cell proliferation [22], highlighting that the neurogenic impact of early isolation depends on precise developmental timing.

Additional evidence shows that isolation has been reported to decrease doublecortin expression—a marker of neuronal immaturity and ongoing neurogenesis [33,34]—indicating reduced numbers of newly generated neurons.

In adult female prairie voles, six weeks of isolation similarly reduced cell proliferation and survival; notably, this reduction extended beyond the dentate gyrus to include the amygdala, medial preoptic area, and ventromedial hypothalamus [35].

In contrast, several studies indicate that social or group housing can buffer the impact of chronic stress on hippocampal neurogenesis. Social housing reduces or prevents stress-induced decreases in BrdU-labeled cells in the dentate gyrus [36], and social enrichment following a period of isolation restores hippocampal BDNF and NGF expression as well as neurogenesis in the dentate gyrus [37]. Additionally, chronic social isolation reduces hippocampal BDNF levels relative to pair-housed rats [38], supporting the notion that group living may exert a neuroprotective effect through neurotrophic mechanisms.

Galea et al. [29] exposed male and female adult rats to an electric-shock stress paradigm under two housing conditions: isolated or group-housed. A reduction in BrdU-labeled cells was observed in isolated animals, whereas group-housed rats showed increased cell proliferation; importantly, sex differences emerged, indicating modulation by gonadal hormones. These changes were not evident in the subventricular zone, even though exposure to a social olfactory stimulus can mitigate stress effects in the dentate gyrus [19]. Social interaction also increases the expression of neurotrophic factors such as brain-derived neurotrophic factor (BDNF), its TrkB receptor [39], and nerve growth factor (NGF) [40], all of which play essential roles in neurogenesis.

Importantly, hippocampal responses to isolation are anatomically dissociable. The dorsal dentate gyrus (dDG) is primarily involved in spatial and contextual memory, whereas the ventral dentate gyrus (vDG) regulates stress, social behavior, and emotional processing. Adult neurogenesis in the vDG has been shown to confer resilience to chronic stress by inhibiting stress-responsive granule cells, whereas suppression of neurogenesis in this region increases stress susceptibility [41]. Emerging data further indicate that social isolation produces selective reductions in cell proliferation in the vDG (but not the dDG), at least in female rodents, following adolescent isolation; these changes persist into adulthood and are not reversed by a resocialization period [42]. These findings suggest that many of the behavioral and affective consequences of isolation likely involve vDG-mediated circuits rather than dorsal hippocampal pathways.

**Figure 2:**
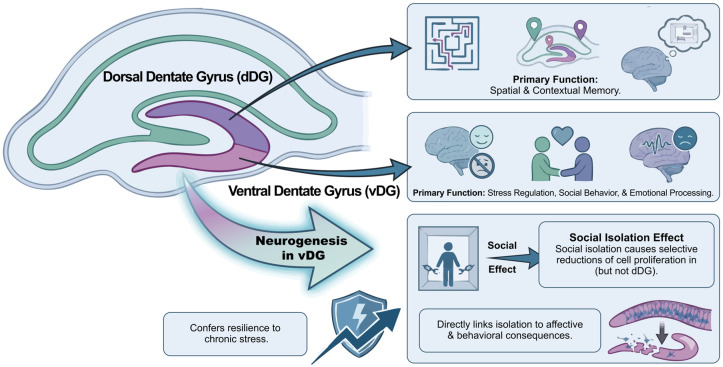
Functional and anatomical dissociation of dorsal and ventral dentate gyrus in social isolation. Conceptual schematic highlighting differential involvement of dorsal and ventral dentate gyrus in cognitive versus affective responses to social isolation. dDG, dorsal dentate gyrus; vDG, ventral dentate gyrus.

These findings indicate that the effects of social isolation on hippocampal neurogenesis are not uniformly distributed along the dentate gyrus. Instead, isolation appears to preferentially affect the ventral dentate gyrus—a subregion critically involved in stress regulation, emotional processing, and social behavior—while sparing the dorsal dentate gyrus, which primarily supports spatial and contextual memory. This functional–anatomical dissociation provides a potential mechanistic link between reduced neurogenesis and the affective and behavioral consequences of isolation (Figure 2).

Moreover, longer or repeated periods of isolation appear more likely to yield persistent structural or neurogenic alterations, consistent with reports showing that even intermediate isolation protocols (e.g., ~14 days) can produce measurable reductions in hippocampal neurogenesis and associated behavioral changes in adult rodents [43].

Table 1 provides an overview of empirical findings describing the behavioral and neurobiological consequences of social isolation across early life, adolescence, and adulthood in both animal models and human populations. Early-life isolation is associated with long-term anxiety-like phenotypes and broad neuroendocrine and neurochemical disruptions [44]. Adolescence represents a particularly sensitive window, with chronic isolation yielding persistent alterations in reward-related behavior, anxiety-like responses, ethanol consumption, and fear extinction [45,46]. In early adulthood, longitudinal analyses indicate that loneliness or social isolation significantly predict depressive episodes and deteriorated mental health [47]. Isolation initiated in adulthood induces limited but measurable behavioral effects alongside transcriptomic changes in monoaminergic pathways [48], whereas long-term isolation leads to neuroendocrine and behavioral alterations in rodent models [49]. Human data from short-term quarantine further support these associations, showing elevated anxiety and depressive symptoms influenced by behavioral and sociodemographic factors [50].

## 4. Social Hierarchy

Systematic evidence in rodents suggests that social status-related paradigms can modulate adult hippocampal neurogenesis, but effects depend strongly on how “hierarchy” is operationalized. The review notes that tube-test outcomes are not consistently associated with neurogenesis changes unless social dominance is explicitly established, whereas paradigms involving dominance interactions and/or aggressive encounters, as well as repeated social defeat, more reliably map onto changes in dentate gyrus neurogenesis markers [11]. The authors emphasize variability across paradigms and outcomes, and reiterate the importance of anchoring interpretations in direct neurogenesis measures (proliferation, immature neuron markers, survival) rather than inferring neurogenic effects from social behavior alone [11].

Dominance or hierarchy stress refers to chronic subordination or repeated social defeat within established social hierarchies, which differs mechanistically from simple isolation or deprivation paradigms.

It is well documented that social groups exhibit a distribution of roles that emerges from competition for limited resources. Dominance and submission have been studied using several paradigms, including the visible burrow system [51]. Dominance not only confers priority access to food, mates, and territory but is also associated with measurable neural differences. Kozorovitskiy and Gould [52] reported increased production of new neurons in the dentate gyrus of dominant male rats compared to subordinates; however, there is no evidence of neurogenesis suppression in subordinate individuals [53,54,55]. This suggests that hierarchy-related variation in neurogenesis depends on circulating hormone concentrations. For example, dominant rats exhibit high levels of testosterone and luteinizing hormone [56], whereas subordinate rats show elevated corticosteroid levels [51,56].

Behaviorally, subordinate rats display reduced aggression and decreased locomotor, sexual, and social activity [57], which may limit their engagement in behaviors that themselves promote neurogenesis, such as sexual interaction. Notably, increased adult neurogenesis has also been observed in dominant baboons compared with subordinates, even when both have access to sexual interaction [58], indicating a specific effect of hierarchical status independent of mating opportunities.

Finally, disrupting an established social status produces divergent effects. Subordinate naked mole-rats, when removed from their colony and isolated, show increased proliferation in the ventral dentate gyrus—likely reflecting the release of social and reproductive suppression [59]. In contrast, disruption of dominant status in other social species is associated with stress-related reductions in hippocampal plasticity, including decreased neurogenesis and altered expression of neuromodulatory systems involved in social behavior. Although specific effects vary across taxa, loss of dominance has been proposed to engage glucocorticoid-dependent mechanisms that may suppress neuronal survival and remodel limbic circuits supporting social and emotional regulation.

Repeated social defeat in rodents results in robust and persistent social avoidance and, in susceptible mice, reduced sucrose preference indicative of anhedonia. Chronic defeat also induces anxiety-like behaviors. Molecular profiling reveals marked transcriptional adaptations in mesolimbic reward regions (NAc, VTA) and the medial prefrontal cortex (mPFC) that sharply differentiate susceptible from resilient animals. These circuit-level and molecular changes have been consistently observed across studies using the standardized social defeat paradigm [60,61]. Pharmacological studies further show that glutamatergic NMDA signaling in the basolateral amygdala is required for both the acquisition and expression of conditioned defeat, whereas GABA_A_-mediated inactivation of the ventromedial prefrontal cortex abolishes dominance-related resistance to defeat [62,63].

In contrast, adolescent social instability stress—brief daily isolation paired with frequent changes of cage partners—produces more subtle yet enduring alterations in social behavior, accompanied by subregion-specific dendritic and synaptic remodeling within the medial amygdala and lateral septum [64]. This paradigm also induces pronounced sex-dependent changes in the neuroendocrine–immune–gut axis, with females showing the strongest alterations in inflammatory signaling, microbial composition, and stress responsivity [65]. Finally, single-unit recordings indicate that anxiety-related firing patterns in the mPFC are tightly coupled to ventral hippocampal input during exploration of anxiogenic environments, highlighting a broader vHPC–mPFC–amygdala circuit through which both defeat and social instability stress may bias emotional processing and social behavior [66].

In parallel to behavioral and neurogenic differences, social hierarchy is also shaped by hormonal and molecular mechanisms that modulate plasticity within stress- and reward-related circuits. Dominant males exhibit elevated testosterone and luteinizing hormone, whereas subordinate individuals show sustained increases in corticosterone [51,56]—a profile consistent with divergent engagement of androgen-responsive pathways that promote neuronal survival in the dentate gyrus [67,68] versus glucocorticoid-dependent suppression of plasticity under chronic subordination [69]. At the molecular level, hierarchy-related differences in neurotrophic and transcriptional signaling have been described across social stress paradigms. Chronic social defeat produces robust remodeling of mesolimbic circuits, including differential activation of BDNF- and CREB-related pathways in susceptible versus resilient animals [39,60,61]. These adaptations intersect with glucocorticoid-receptor-dependent mechanisms known to regulate stress responsivity and long-term behavioral outcomes [69]. Together, these endocrine and molecular mechanisms provide a coherent framework linking hierarchical status to stress vulnerability, circuit plasticity, and individual differences in resilience.

## 5. Social Buffering

In this section, “social buffering” refers specifically to the attenuation of physiological or behavioral stress responses in the presence of a conspecific during or immediately following a stressor. This construct is distinguished from general social support or co-housing conditions not explicitly paired with stress exposure.

A recent multisystem meta-analysis in rats quantitatively benchmarks the magnitude and consistency of social buffering effects across behavioral and physiological stress readouts, and proposes a translational framework for stress resilience grounded in convergent endpoints rather than single-marker inference [70]. Importantly, this synthesis highlights that buffering effects are not uniform across all outcomes or paradigms, and that heterogeneity in stressor type, timing, and social context can moderate effect direction and magnitude. We therefore treat social buffering as a graded, context-dependent phenomenon and interpret putative neurogenic links as plausible but not universally demonstrated unless cellular endpoints are concurrently measured.

Across development and species, supportive social partners have been shown to dampen HPA-axis activity and reduce cortisol/corticosterone output [71,72]. In rodents, the presence of an adult conspecific during threat exposure markedly suppresses freezing and attenuates corticosterone responses, demonstrating that social cues buffer both behavioral and endocrine components of the stress reaction [73,74]. Human studies similarly show that attachment figures reduce cortisol reactivity to social-evaluative stress, indicating an evolutionarily conserved mechanism for regulating HPA-axis responsivity [71].

**Figure 3:**
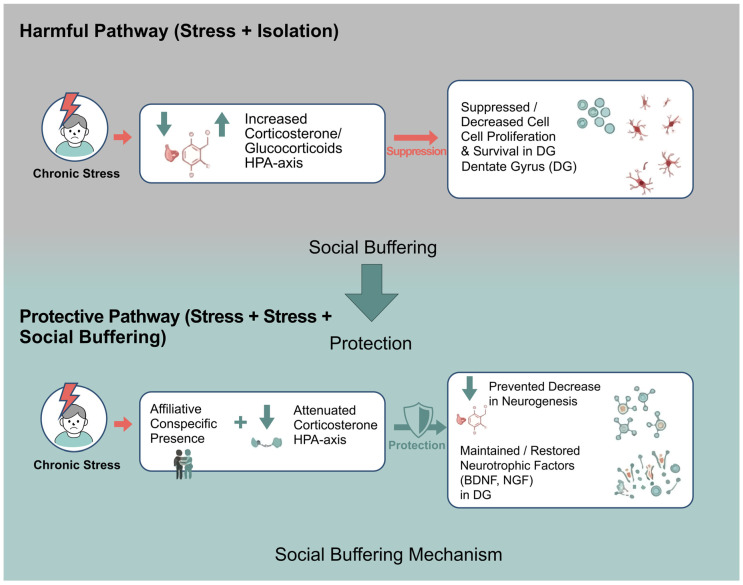
Social buffering as a conceptual framework for attenuating stress-related suppression of neurogenesis. Conceptual overview illustrating how affiliative social interactions attenuate stress-hormone signaling and may help preserve hippocampal neurogenesis when directly quantified. DG, dentate gyrus; HPA, hypothalamic–pituitary–adrenal axis.

At the mechanistic level, social buffering operates by dampening stress-hormone signaling and preserving plasticity within stress-sensitive hippocampal circuits. In the absence of affiliative social contact, chronic stress leads to sustained activation of the HPA axis, elevated glucocorticoids, and suppression of cell proliferation and survival in the dentate gyrus. By contrast, the presence of an affiliative conspecific has been shown to attenuate corticosterone responses, maintains neurotrophic support, and has been associated with attenuation of stress-related suppression of hippocampal neurogenesis in paradigms that directly quantify cellular endpoints (Figure 3).

At the neural and plasticity level, social buffering mitigates the deleterious effects of chronic stress on mesolimbic and hippocampal circuits. Rewarding social interaction reverses stress-induced behavioral and molecular alterations in limbic regions [17], whereas socially impoverished or unstable environments reduce hippocampal neurogenesis and dysregulate stress-hormone profiles—often in sex-specific ways [30,31,36]. These findings converge with evidence that increased neurogenesis in the ventral dentate gyrus promotes resilience by inhibiting stress-responsive granule cells and limiting pathological engagement of stress circuits [41]. Together, these results position social buffering as a multilevel protective framework—hormonal and neurocircuit-level, with conditional support for neurogenic involvement—that stabilizes HPA-axis output, maintains ventral hippocampal plasticity, and may reduce vulnerability to stress-related psychopathology, particularly in paradigms where cellular neurogenesis endpoints are directly assessed.

## 6. Social Learning & Social Memory

Social learning depends on the ability to acquire information from conspecifics, a process grounded in social recognition memory—the capacity to discriminate familiar from unfamiliar individuals and to encode socially relevant cues. This form of memory is strongly regulated by the neuropeptides oxytocin (OXT) and vasopressin (AVP), which modulate olfactory–limbic circuits supporting identity recognition [75,76]. Extensive reviews show that disrupting OXT/AVP signaling—whether pharmacologically, genetically, or circuit-specifically—reliably impairs social recognition across rodent models, underscoring the central role of these neuropeptide systems in encoding and retrieving social identities [75,76,77].

Within the hippocampal formation, the CA2 subregion and ventral CA1 constitute key nodes of a circuit specialized for social memory. Converging evidence shows that disrupting CA2 function—or its direct input from the lateral entorhinal cortex—selectively impairs the ability of rodents to discriminate familiar from novel conspecifics, while leaving non-social memory domains intact [78,79]. In particular, optogenetic silencing of LEC→CA2 projections abolishes social recognition memory without affecting object recognition, indicating that CA2-dependent computations are specifically tuned to socially relevant cues [79]. As reviewed by Wang & Zhan [78], social memory representations in CA2 and vCA1 interface with broader limbic and motivational circuits—including septal, prefrontal, and nucleus accumbens pathways—that encode social salience, identity, and motivational value, situating CA2 as a central hub within the social information-processing network.

Adult hippocampal neurogenesis provides an additional layer of plasticity for social information processing. Experimental suppression of adult-born granule cells impairs social recognition memory in rodents, indicating that new neurons contribute to the stability, maintenance, and updating of social representations [80]. Conversely, environmental enrichment—which enhances adult neurogenesis—restores social memory deficits induced by previous isolation, and this improvement is abolished when cell proliferation is pharmacologically blocked, supporting a causal role for neurogenesis in social memory persistence [81]. Complementary systematic reviews highlight that social behaviors both influence and depend on neurogenesis within hippocampal and olfactory circuits, integrating adult-born neurons into a broader network supporting social learning [11]. These mechanisms also shape how rodents adapt to socially structured environments, including the formation of stable dominance hierarchies. Dominant males exhibit greater survival of adult-born neurons in the dentate gyrus compared with subordinate males, indicating that hierarchical status modulates hippocampal plasticity. Although this study did not directly examine social memory or identity encoding, adult-born granule cells are broadly implicated in contextual and affective processing, suggesting that neurogenesis may influence how animals navigate complex social situations. However, the specific encoding of individual identity, rank, or social network structure requires evidence from other research domains and is beyond the scope of Kozorovitskiy & Gould [52].

## 7. Sex Differences and Sexual Dimorphism

A comprehensive review of sex differences in hippocampal cognition and adult neurogenesis highlights that net neurogenesis reflects coordinated changes in proliferation, differentiation, and survival, and that sex differences can emerge at specific stages and under specific challenges [82]. Under basal conditions, several studies report sex differences in dentate gyrus cell proliferation (often modulated by estrous phase or season), whereas most studies do not find robust sex differences in survival of new neurons across rodents, with exceptions in some datasets [82]. Importantly, the review emphasizes that neurogenesis responses to stress and hippocampus-dependent learning can be sex-dependent and may interact with learning strategy and hormonal milieu, underscoring sex as a biological variable that can shape both neurogenic dynamics and behavioral readouts [82].

Sex differences and sexual dimorphism shape how mammals integrate social experience, stress, and reproductive demands into neural plasticity and behavior. In this context, sex differences refer to quantitative variations in traits shared by both sexes—such as the magnitude of hormonal responses, levels of neurogenesis, or behavioral intensity—whereas sexual dimorphism denotes qualitative distinctions in phenotype, physiology, or behavior that arise from sex-specific developmental pathways, leading males and females to express partially non-overlapping strategies or neural specializations.

Males and females exhibit distinct neuroendocrine and behavioral responses to social stress. Males often show stress-induced reductions in hippocampal neurogenesis and alterations in defensive behavior, whereas females display sex-specific changes in anxiety, social motivation, and stress responsivity [36,54,83,84]. These dimorphisms extend to parental care, mating, and aggression, where the hormonal milieu modulates hippocampal plasticity and social circuits. In males, androgens enhance adult-born neuron survival and influence sexually motivated behavior and aggression [67,85,86]. In females, neurogenesis is dynamically regulated across pregnancy, lactation, and the postpartum period by estrogens, progesterone, adrenal steroids, and prolactin [87,88,89].

Emerging evidence also indicates that males and females rely on partially distinct neural pathways for processing social information, social memory, and social buffering, reflecting dimorphic organization of corticolimbic and hypothalamic systems [12,28,90].

Collectively, these findings support the growing field of sex-informed neuroscience, which conceptualizes sex as a biological variable that systematically modulates neurogenesis, hormone–brain interactions, stress vulnerability, parental and reproductive strategies, and the adaptive use of social environments across the lifespan.

Importantly, the evidence base synthesized in this review is not evenly distributed across sexes or paradigms. Direct cellular neurogenesis endpoints under social stress have been evaluated in both sexes in some designs (e.g., chronic stress combined with individual vs. social housing), but several stress paradigms remain male-weighted in the literature, whereas reproductive transitions provide necessarily female-specific evidence. In this domain, pregnancy has been linked to increased progenitor production in the forebrain SVZ with downstream addition of olfactory interneurons [87], while postpartum and parity-related effects on adult neurogenesis have been directly quantified in the dentate gyrus, including suppressed proliferation and/or survival in early postpartum depending on reproductive experience [89]. Conversely, androgen-linked modulation of adult-born neuron survival has been characterized primarily in male-focused studies and is expressed mainly at the level of cell survival (not proliferation) via androgen receptor-dependent mechanisms [67]. Studies that explicitly test sex in stress responsivity and hippocampal outcomes further support sex-dependent mechanisms, while also underscoring the need for balanced factorial designs that quantify matched neurogenesis components (proliferation, survival, maturation/integration) in both sexes under comparable social manipulations [28,36,54,90]. Together, these patterns suggest that current conclusions reflect both biological sex-dependent regulation and methodological asymmetries in sampling and endpoint selection, motivating more systematically sex-balanced approaches in future work.

## 8. Parenting

Early-life adversity has been synthesized in a three-level meta-analysis of rodent studies demonstrating that developmental adversity produces robust but heterogeneous behavioral phenotypes, with effect sizes moderated by methodological and biological factors (e.g., type and timing of adversity, sex, and testing domain) [91]. This meta-analytic structure underscores that “early-life adversity” is not a single manipulation and that outcome direction and magnitude vary systematically with moderators rather than reflecting a unitary effect. Accordingly, when we discuss maternal care/adversity in relation to adult neurogenesis, we explicitly separate (i) meta-analytic behavioral evidence, (ii) mechanistic pathway plausibility, and (iii) direct cellular neurogenesis findings, avoiding causal smoothing when these tiers are not measured within the same studies.

Like other social behaviors, parenting is strongly modulated by hormonal changes [84], particularly during pregnancy and throughout contact with the infant, including breastfeeding [12]. However, its effects on neurogenesis vary depending on the specific stage of caregiving. During lactation [92] and the postpartum period [93], elevated corticosterone levels have been reported, suppressing the generation of new neurons and suggesting that the stressful component of parenting can negatively impact hippocampal plasticity.

Conversely, other findings point to positive neuroplastic effects associated with maternity. Leuner and Gould [88] reported increased dendritic spine density in the hippocampus and medial prefrontal cortex, and a buffering effect of motherhood on stress-induced learning deficits has also been described [94]. In addition, some rodent studies report parenting-associated increases in proliferation and/or survival of adult-born neurons [89].

Taken together, these results suggest that parenting exerts bidirectional effects on neurogenesis: positive, reward-related influences hypothesized to relate to hedonic and affiliative components of caregiving, and negative influences associated with the physiological stress inherent to parental demands.

Beyond the general effects of parenting on hippocampal plasticity, important distinctions emerge between maternal and paternal care. In mothers, pregnancy, lactation, and infant contact produce marked fluctuations in estrogens, progesterone, oxytocin, and prolactin—a hormone that directly stimulates neural progenitor proliferation in the subventricular zone and facilitates maternal behavioral responsiveness [87,95].

In fathers, parenting induces different forms of neural plasticity: paternal experience suppresses adult hippocampal neurogenesis in Peromyscus californicus, whereas fatherhood increases dendritic spine density in dentate gyrus granule cells and CA1 pyramidal neurons and reduces anxiety-like behavior—changes that reflect circuit-level adaptations supporting paternal care [96,97,98].

Human EEG and neuroimaging studies similarly show that the postpartum period involves experience-dependent changes in prefrontal–parietal synchronization and reward-related activity when mothers process infant cues, revealing cortical plasticity that enhances sensitivity to infant signals [99,100].

Taken together, parenting integrates both a stress component—reflected in corticosterone-associated suppression of neurogenesis—and a rewarding component mediated by prolactin-, oxytocin-, and dopamine-related mechanisms that promote attachment and motivation [12,94,101]. The interplay between these stress-related and hedonic pathways determines the direction and magnitude of plastic changes across hippocampal and prefrontal systems during the parental period.

To provide a structured synthesis of parental-stage-dependent plasticity, Table 2 reorganizes the evidence according to species, sex, reproductive stage, and specific caregiving context, explicitly differentiating direct adult neurogenesis measurements from indirect structural or functional adaptations. Where available, neurogenesis endpoints are specified by component (e.g., proliferation or survival) and brain region (SVZ or dentate gyrus), along with the reported direction of effect. Human neuroimaging and circuit-level studies are presented separately as indirect evidence of structural or functional plasticity. This organization clarifies which parental adaptations are supported by direct cellular markers and which reflect broader neuroendocrine or network-level remodeling, thereby reducing interpretive overextension while preserving the developmental logic of caregiving-related brain plasticity.

## 9. Sexual Interaction

An integrative review on the regulation of adult hippocampal neurogenesis by sexual, cognitive, and physical activity summarizes evidence that sexual experience can increase dentate gyrus neurogenesis, including after single or repeated exposure in middle-aged rodents, and that longer-term repeated access may coincide with improvements in cognitive performance while mating persists [102]. The authors highlight the motivational context of sexual behavior as a reward state that may promote repeated engagement, and they note that repeated sexual experience has been proposed to promote incorporation of new neurons and to protect against stress-related suppression of neurogenesis in some paradigms [102]. They also emphasize translational limits, noting that there are no human data directly demonstrating that sexual behavior induces hippocampal neurogenesis [102].

Sexual interaction is a potent social reinforcer that engages motivational and reward circuits while producing hormone-dependent effects on adult hippocampal plasticity. In rodents and other mammals, copulation and sexually relevant cues recruit mesolimbic dopamine pathways—particularly within the nucleus accumbens—in ways that parallel other natural rewards. Dopaminergic signaling contributes to the incentive value of sexual stimuli and to the formation of conditioned partner and place preferences, even though dopamine is not required for the motor execution of copulation itself [103,104]. Through these mechanisms, sexual interaction influences not only reproductive outcomes but also the motivational and affective components of social behavior.

In females, estradiol has been shown to rapidly increase proliferation on neural progenitors in the dentate gyrus. Estradiol surges—whether naturally occurring during proestrus or experimentally induced—transiently increase the number of dividing hippocampal cells [105,106]. These effects fluctuate across the estrous cycle, with proestrus/high-estradiol phases enhancing both sexual receptivity and hippocampal cell proliferation. Because estradiol also facilitates appetitive aspects of sexual behavior and modulates dopaminergic responses to sexual stimuli, estrous-cycle variation likely coordinates reward-circuit recruitment with periods of heightened neurogenic plasticity [103,107].

In males, testosterone has been shown to influence the survival and maturation of adult-born granule neurons. Experimental studies suggest that testosterone and dihydrotestosterone increase the survival of newly generated neurons through androgen receptor-dependent mechanisms [67,86]. Sexual activity elevates testosterone and engages these pathways, potentially linking mating to long-term changes in hippocampal circuit integration [85]. Thus, estradiol predominantly influences proliferative phases in females, whereas testosterone modulates survival and functional incorporation of new neurons in males.

The integrity of gonadal hormone signaling appears critical for the neuroplastic and motivational effects of sexual interaction. Peripubertal social isolation has been shown to reduce adult testosterone levels and weaken sexually motivated behavior without necessarily impairing copulatory performance [26,27]. Moreover, the typical association between testosterone fluctuations and limbic theta activity observed in socially housed males is disrupted following adolescent isolation [27]. These findings indicate that early social environment may shape later neuroendocrine responsiveness to sexual stimuli, potentially influencing how reproductive behaviors interface with adult plasticity mechanisms.

Sexual experience has been associated with changes in stress reactivity and emotional resilience. Although mating can acutely elevate glucocorticoids, repeated sexual interaction has been reported to increase hippocampal neurogenesis in some rodent paradigms, and to coincide with improvements in anxiety- and stress-related behaviors [88]. These effects likely arise from interactions among mesolimbic dopamine, gonadal steroids, and hippocampal plasticity mechanisms. Sexual reward recruits neuromodulatory systems that can buffer stress-related suppression of neurogenesis and promote adaptive emotional regulation [12].

Collectively, sexual interaction influences adult neurogenesis through four coordinated mechanisms:

1. recruitment of mesolimbic dopamine circuits as a socially rewarding stimulus;
2. estradiol-dependent increases in progenitor proliferation across the estrous cycle;
3. testosterone-dependent enhancement of new-neuron survival; and
4. experience-driven modulation of reward and stress pathways that jointly support adaptive hippocampal plasticity.

## 10. Neurochemical Pathways of Socialization

A recent mechanistic synthesis proposes that adult hippocampal neurogenesis may bias hippocampal computations toward enhanced contextual discrimination (pattern separation) and adaptive stress regulation, thereby shaping downstream behavioral and neuroendocrine responses [108]. The authors argue that adult-born neurons are not necessarily required for all baseline hippocampal functions, but may fine-tune specific processes relevant to stress adaptation and memory precision, offering a useful framework for interpreting how stress- and social-interaction–engaged pathways could converge on neurogenic regulation [108].

As noted above, diverse forms of social behavior influence adult neurogenesis through multiple physiological, hormonal, and neurochemical pathways. These effects are particularly evident in the two principal neurogenic niches of the adult brain—the subventricular zone (SVZ), which supplies newborn neurons to the olfactory bulb, and the dentate gyrus of the hippocampus [109,110]. Socially induced changes in neurogenesis are broadly regulated by neuroendocrine systems and by the synthesis, release, and receptor-mediated actions of several neuromodulators and trophic factors.

Social interaction can be either reinforcing or aversive, and correspondingly may promote or suppress neurogenesis. These bidirectional effects are largely orchestrated by hormones of the hypothalamic–pituitary–adrenal (HPA) and hypothalamic–pituitary–gonadal (HPG) axes [29]. Reinforcing social experiences—such as mating, bonding, or parental contact— are associated with activation of gonadal-steroid systems that has been proposed to facilitate neurogenic processes, whereas aversive experiences, including social defeat or chronic isolation, activate glucocorticoid pathways that inhibit neurogenesis.

Gonadal hormones exert particularly strong and dynamic influences on neural progenitor proliferation and survival. Estradiol is closely associated with increased production of new neurons [12]. Acute estradiol administration stimulates hippocampal cell proliferation within 2–4 h, although this effect disappears by 48 h [111]. However, high-dose estradiol [105,112] or chronic administration at any dose does not enhance proliferation [107]. These suppressive outcomes observed at elevated estradiol levels appear to be mediated by glucocorticoids [113]. Moreover, estradiol’s effects on neurogenesis and dendritic spine density vary as a function of sex [114], age, and reproductive status [115]. For instance, females in proestrus display higher rates of hippocampal cell proliferation than males [106], although this pattern is not preserved across all species. The hippocampus contains abundant receptors for gonadal steroids, oxytocin, luteinizing hormone (LH), and prolactin—hormones strongly implicated in sexual behavior and parental care [29]. Prolactin receptors are present in both the SVZ and the hippocampus [116]. Exposure to pheromones from a dominant male increases cell proliferation in the olfactory bulb and hippocampus of female mice [95], while pregnancy induces pronounced proliferation in the SVZ [87]. These effects appear to be mediated by prolactin in the olfactory bulb and by LH in the hippocampus [117]. Additionally, prolactin may confer neuroprotective effects during chronic stress. Notably, however, early postnatal administration of prolactin (postnatal day 14) reduces neurogenesis, indicating that the hormone’s effects are strongly age-dependent.

**Figure 4:**
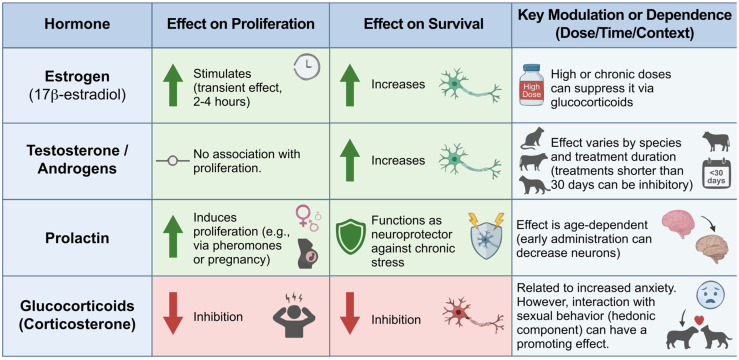
Hormonal modulation of adult neurogenesis. Schematic overview of hormone-specific effects on neurogenic stages under different physiological contexts.

Adult neurogenesis is tightly regulated by endocrine signals that differentially influence distinct stages of the neurogenic process. Gonadal hormones, prolactin, and glucocorticoids exert stage-specific and context-dependent effects on progenitor proliferation and neuronal survival, with outcomes shaped by dose, duration of exposure, developmental stage, and behavioral state. A comparative overview of these hormone-specific actions is provided in Figure 4.

Beyond hormonal and classical neurochemical pathways, adverse social experiences also have been shown to involve immune–glial mechanisms that modulate adult neurogenesis. Long-term social isolation in middle-aged mice decreases ΔFosB expression in the dentate gyrus and reduces BDNF levels in CA3, without increasing hippocampal oxidative damage. These findings indicate a state of reduced neuronal activation and diminished trophic support that likely contributes to impaired plasticity [49]. Shorter isolation periods in adult rats induce transcriptional reorganization of monoaminergic and peptidergic signaling in the medial prefrontal cortex—downregulating RGS9, HTR2C, and Pdyn, among others—alongside measurable alterations in social behavior, demonstrating that isolation stress rapidly reshapes prefrontal neuromodulatory dynamics [48]. Complementary evidence from chronic social defeat shows that repeated social stress suppresses cell proliferation in the dentate gyrus and elicits robust microglial activation in hippocampal and prefrontal regions, including hypertrophic, cytokine-expressing phenotypes [118]. Taken together, these findings suggest that microglial and inflammatory processes—acting in concert with monoaminergic and neurotrophic changes—may represent a critical interface through which adverse social environments remodel synaptic function and constrain adult neurogenic plasticity.

Estrogens influence every stage of neurogenesis—including proliferation, differentiation, migration, and maturation. Administration of selective agonists for the alpha (ERα) and beta (ERβ) estrogen receptors regulates cell proliferation in adult female rats [119]. The survival of newly generated neurons depends on the type of estrogen administered, the timing of administration, and the behavioral context in which it occurs. For instance, administration of 17β-estradiol (the most potent endogenous estrogen) increases the survival of newborn neurons in rats performing a maze task, whereas administration of estrone decreases neuronal survival [120].

Androgens constitute another major class of gonadal hormones influencing adult neurogenesis, and their levels increase following sexual interaction [85]. The dentate gyrus as well as the CA1 and CA3 subfields of the hippocampus contain androgen receptors [67], which mediate their specific neurogenic effects. Testosterone and its metabolite dihydrotestosterone enhance the survival of newly generated neurons but do not increase proliferation [12]. Conversely, castrated rats exhibit reduced neuronal survival [86], while adolescent macaques that underwent gonadectomy show increased hippocampal neuronal survival [121]. These findings indicate that, similar to estrogens, androgen effects vary across species, age, sex, and treatment duration. For example, testosterone treatment lasting fewer than 30 days has been associated with an inhibitory effect on neuronal survival [122].

Hormones of the hypothalamic–pituitary–adrenal (HPA) axis also regulate stress effects on neurogenesis and interact bidirectionally with hormones of the hypothalamic–pituitary–gonadal (HPG) axis. Spritzer et al. [68] reported that castrated adult male rats show fewer dentate gyrus cells after one month of isolation compared with intact males. Increased glucocorticoid levels are typically associated with reduced cell proliferation and heightened anxiety [69,123], as well as elevated corticosterone and estradiol levels during aging [113]. However, elevated glucocorticoids do not invariably produce negative effects on neurogenesis; for example, the interaction between sexual activity and stress can promote neurogenesis despite increased glucocorticoid release—a phenomenon likely driven by the hedonic component of sexual interaction [12]. As with other modulators of neurogenesis, these effects are not unidirectional: outcomes depend on complex interactions among hormonal, environmental, and individual variables.

**Figure 5:**
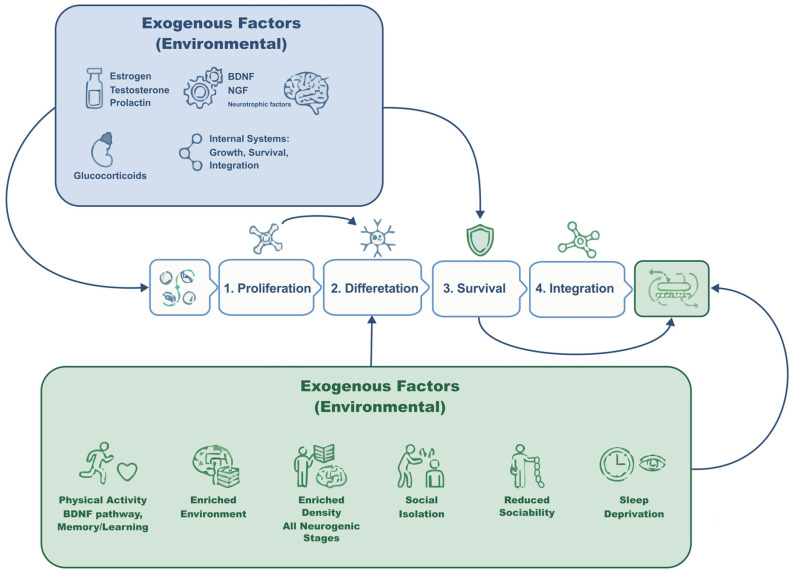
Social and endocrine modulation of adult neurogenesis across neurogenic stages. Adult neurogenesis proceeds through sequential stages of proliferation, differentiation, survival, and functional integration, which are dynamically regulated by internal endocrine systems and external environmental factors. Social experiences can either promote or suppress neurogenic plasticity by engaging gonadal hormones, neurotrophic signaling, or stress-related glucocorticoid pathways, thereby shaping hippocampal and subventricular neurogenesis in a context-dependent manner. DG, dentate gyrus; SVZ, subventricular zone; BDNF, brain-derived neurotrophic factor; NGF, nerve growth factor.

Together, these findings indicate that social experience does not act on adult neurogenesis through a single pathway, but rather through coordinated endocrine, neurochemical, and environmental mechanisms that converge on distinct stages of the neurogenic process. Socially reinforcing behaviors, such as mating, affiliation, and parental interaction, preferentially engage gonadal hormones and neurotrophic signaling to promote neuronal survival and integration, whereas aversive conditions—including chronic isolation and social stress—activate glucocorticoid-dependent pathways that suppress proliferation and impair maturation. Importantly, these effects are stage-specific and context-dependent, highlighting adult neurogenesis as a dynamic interface between social experience and brain plasticity (Figure 5).

To provide a structured synthesis of the hormonal and neurochemical systems discussed above, Table 3 reorganizes the evidence according to species, experimental context, specific adult neurogenesis endpoints, and brain region. Where available, studies directly measuring proliferation or survival (e.g., BrdU-based assays in the dentate gyrus or subventricular zone) are distinguished from indirect mechanistic or synaptic findings that do not include cellular neurogenesis markers. This differentiation clarifies which endocrine signals (e.g., estradiol, androgens, prolactin) have been shown to modulate defined components of adult neurogenesis, and which systems (e.g., oxytocin, BDNF, monoaminergic and stress-related pathways) are supported primarily by circuit-level, molecular, or review-based evidence. By explicitly specifying endpoint type, anatomical region, and direction of reported effects, the table aims to reduce overgeneralization while preserving the functional relevance of these systems to social behavior.

## 11. Critical Considerations and Conceptual Gaps

While the present review synthesizes converging evidence linking social experience and adult neurogenesis, several conceptual considerations merit attention.

First, much of the available literature derives from rodent models, and the magnitude and direction of neurogenic effects appear to vary across species, sexes, and social contexts. This variability suggests that social modulation of neurogenesis is contingent rather than uniform.

Second, a substantial portion of the evidence is correlational or context-dependent, as social manipulations often co-occur with alterations in stress physiology, metabolic state, or environmental complexity. Disentangling these interacting variables remains an important methodological challenge.

Third, the directionality of the relationship remains incompletely resolved. While social experiences influence neurogenic processes, hippocampal plasticity may also shape social behavior, raising the possibility of bidirectional regulation.

Addressing these issues will be essential for refining translational interpretations and clarifying the adaptive significance of socially regulated neurogenesis across species.

### 11.1. Evidence Stratification and Causal Boundaries

The literature reviewed integrates findings from multiple levels of analysis, which differ substantially in evidentiary strength. First, direct adult neurogenesis evidence derives from studies quantifying cellular endpoints (e.g., BrdU, Ki67, DCX) in the dentate gyrus or subventricular zone, allowing conclusions regarding proliferation, survival, or maturation. Second, mechanistic studies examining oxytocinergic, glucocorticoid, inflammatory, or monoaminergic pathways frequently do not include concurrent neurogenesis markers and therefore provide pathway-level plausibility rather than direct demonstration of neurogenic modulation. Third, behavioral outcomes—such as social avoidance, dominance-related stress phenotypes, or parental responsiveness—are functionally relevant but do not constitute evidence of altered adult neurogenesis unless cellular measures are included.

Accordingly, causal interpretations should be graded. In several domains, links between social experience and adult neurogenesis remain hypothesis-driven or context-dependent rather than definitively established. Recognizing these distinctions prevents artificial smoothing of causal chains and highlights the need for integrative designs combining cellular, mechanistic, and behavioral endpoints within the same experimental framework.

### 11.2. Methodological Confounds and Unit-of-Analysis Constraints

Interpretation of socially induced neurogenic changes is further complicated by methodological confounds inherent to many experimental paradigms. Social manipulations frequently co-occur with alterations in stress intensity, environmental enrichment, locomotor activity, sleep patterns, metabolic state, and sensory stimulation. As a result, observed changes in proliferation or survival may reflect composite physiological adaptations rather than isolated effects of a single social variable. Disentangling deprivation, threat, affiliative buffering, and enrichment components remains a central challenge in the field.

In addition, housing-based paradigms raise concerns regarding the appropriate unit of analysis. In many social isolation or group-housing designs, the experimental manipulation is applied at the cage level, whereas statistical analyses are often conducted at the individual animal level. When clustering effects are not explicitly accounted for, this may inflate statistical power and overestimate effect robustness. These design considerations directly influence strength-of-evidence grading and should be considered when interpreting reported neurogenic outcomes.

Greater methodological precision—including multilevel modeling approaches, explicit reporting of clustering, and factorial designs separating social, stress-related, and enrichment variables—will be essential for strengthening causal inference in future studies.

## 12. Comparative Models

Comparative models across mammals, birds, and other vertebrates provide crucial insights into how social structure, mating systems, and communication demands shape adult neurogenesis. Species such as prairie voles, naked mole-rats, nonhuman primates, and songbirds illustrate the diversity of neural adaptations supporting monogamy, eusociality, hierarchical organization, and vocal-based social learning. These models reveal that sociality can either amplify or suppress neurogenic activity depending on ecological pressures and behavioral specialization, offering an evolutionary lens through which to interpret rodent findings.

Comparative species models—including socially monogamous prairie voles, eusocial mole-rats, nonhuman primates, songbirds, and teleost fish—provide a framework for examining how distinct forms of social organization relate to adult neurogenesis across taxa. As summarized in Table 4, some models offer direct experimental evidence that specific social contexts (e.g., pair bonding, social valence, seasonal communication) modulate defined neurogenic endpoints, whereas others provide comparative or descriptive data on baseline neurogenesis rates under different ecological or social structures. By explicitly distinguishing experimentally demonstrated modulation from interspecies variation, the table clarifies which associations are supported by direct cellular measurements and which remain ecological or translational in nature.

## 13. Conclusions and Future Directions

Across the evidence reviewed, social experience emerges as a significant modulator of adult neurogenesis, influencing every stage of the neurogenic trajectory—from progenitor proliferation to neuronal survival and functional integration. Social interaction, parenting, sexual behavior, hierarchy formation, and group living have been shown in multiple paradigms to modulate neurogenesis through coordinated endocrine and neurochemical pathways involving gonadal and adrenal hormones, oxytocin- and vasopressin-dependent systems, neurotrophic signaling, and monoaminergic modulation. Conversely, adverse conditions such as social isolation or chronic social stress have been consistently associated with reductions in hippocampal plasticity, particularly within ventral dentate gyrus circuits that govern emotional regulation and stress responsivity.

A core conclusion is that the neurogenic impact of social behavior is highly context-dependent, shaped by developmental stage, sex, hormonal milieu, species-specific ecology, and the duration and valence (reinforcing vs. aversive) of the social experience. Parenting illustrates this duality: prolactin-, oxytocin-, and reward-related mechanisms can enhance progenitor activity, whereas corticosterone elevations during stressful caregiving demands suppress hippocampal neurogenesis. Similarly, hierarchy formation and social status modulate neurogenesis through contrasting hormonal profiles, with dominant individuals often showing enhanced neuronal survival.

Comparative models reinforce this diversity. Socially monogamous voles, eusocial mole-rats, primates with complex hierarchies, songbirds with vocal learning systems, and teleost fish with high regenerative capacity demonstrate that sociality can either amplify or constrain neurogenesis depending on evolutionary pressures and behavioral specialization. These species illustrate that adult neurogenesis is not a monolithic phenomenon, but a plastic process embedded within ecological and social adaptations.

Future work should refine how specific social behaviors and hormonal contexts are associated with distinct neurogenic outcomes across development, sex, and species, particularly in paradigms where current evidence remains limited or context-dependent. Integrating cellular endpoints (proliferation, survival, maturation), endocrine dynamics, and circuit-level analyses within the same experimental frameworks will be important for determining when socially related changes in neurogenesis are directly demonstrated versus mechanistically inferred. As synthesized in the revised tables, the available data suggest that social experience can modulate adult neurogenesis under specific biological and environmental conditions; however, the magnitude, direction, and functional significance of these effects vary across paradigms and species. A graded interpretation of this literature therefore suggests cautious but convergent evidence that socially relevant contexts interact with neurogenic processes, while underscoring the need for balanced, factorial, and translationally disciplined designs.

## Figures and Tables

**Table 1:** Behavioral and Neurobiological Effects of Social Isolation Across Developmental Stages.

Species/Model	Sex	Developmental Window	Isolation Protocol	Primary Endpoint Assessed	Main Findings	Reference
Rat (Sprague–Dawley)	Male	Post-weaning → adulthood	Early-life social isolation	Behavioral (anxiety-like tests); neuroendocrine & monoaminergic markers	Increased anxiety-like behavior; altered oxytocin, melatonin, ghrelin, and monoamine levels	[44]
Rat (Sprague–Dawley)	Female	Adolescence (P30–50)	Chronic isolation	Behavioral (forced swim; sucrose preference)	Altered stress-coping and reward-related behavior (sex-specific)	[46]
Rat (Long–Evans)	Male	Adolescence	Chronic isolation	Behavioral (anxiety tests; ethanol intake; fear extinction)	Increased anxiety-like behavior and ethanol intake; impaired fear extinction	[45]
Rat	Male	Adulthood (10 days)	Short-term social isolation	Molecular (RNA-seq; monoaminergic signaling genes)	Differential expression of monoaminergic-related genes in mPFC	[48]
Mouse (C57BL/6)	Male	Adulthood (30 weeks)	Long-term single housing	Molecular (ΔFosB; BDNF); Behavioral (memory)	Reduced ΔFosB in dentate gyrus; reduced BDNF in CA3; memory impairment	[49]
Human (university students)	Male & Female	Young adulthood (longitudinal)	Loneliness exposure	Epidemiological (diagnostic outcomes)	Increased risk of major depressive episodes over time	[47]
Human (COVID-19 cohort)	Male & Female	Adulthood	Mandatory confinement	Epidemiological (symptom scales)	Increased anxiety and depressive symptom severity	[50]

None of the studies summarized in this table directly assessed adult neurogenesis; findings represent behavioral, molecular, or epidemiological evidence associated with social isolation. Abbreviations: mPFC, medial prefrontal cortex; ΔFosB, truncated FosB transcription factor; BDNF, brain-derived neurotrophic factor; CA3, cornu ammonis 3 (hippocampal subfield).

**Table 2:** Endocrine and neurobiological changes across stages of parental behavior.

Species/Strain	Sex	Reproductive Stage/Manipulation	Social Manipulation Characteristics	Neurogenesis Readout	Component	Brain Region	Direction	Evidence Type	Evidence Strength	Reference
Mouse (CD1)	Female	Pregnancy (GD7)	Natural pregnancy; BrdU labeling	BrdU, Ki67	Proliferation	SVZ	↑	Direct	Limited (single primary study)	[87]
Rat (Wistar)	Female	Lactation (LD2–LD13)	Natural lactation ± repeated restraint stress	BrdU; cell survival; differentiation	Proliferation ↓ (baseline lactation); stress reverses ↓	Dentate gyrus	↓ (baseline); normalization with stress	Direct	Limited–mixed (condition-dependent)	[93]
Rodent (review synthesis)	Female	Peripartum	Endogenous prolactin elevations	SVZ & hippocampal neurogenesis (reviewed evidence)	Proliferation & survival	SVZ; DG	Context-dependent	Direct + mechanistic	Mixed	[95]
California mouse (Peromyscus californicus)	Male	Paternal experience	Cohabitation with mate and offspring	BrdU	Proliferation/survival	Dentate gyrus	↓	Direct	Limited (species-specific)	[97]
California mouse	Male	Fatherhood	Natural paternal experience	Dendritic spine density (not neurogenesis)	Structural plasticity	Hippocampus	↑ spine density	Indirect (structural)	Limited	[98]
Human	Female	Early postpartum (2–4 wks to 3–4 months)	Longitudinal MRI	VBM gray matter volume	Structural volume	PFC, parietal, midbrain	↑ GM volume	Indirect (no neurogenesis measure)	Limited	[101]
Human	Female	Postpartum	Infant-cue exposure during fMRI	Functional synchronization	Network activity	PFC–parietal	Modulation	Indirect	Limited	[100]
Human (review)	Female	Peripartum	Hormonal transition	Neurobiological adaptations	Conceptual	Multiple regions	Context-dependent	Indirect	Narrative synthesis	[99]
Rodent (review synthesis)	Female	Parenting	Hormonal shifts (prolactin, oxytocin)	DG neurogenesis (reviewed)	Proliferation/survival	Dentate gyrus	Mixed	Direct + mechanistic	Mixed	[12]

Evidence type: Direct = neurogenesis markers measured (BrdU, Ki67, survival, differentiation); Indirect = structural MRI, dendritic morphology, network activity without neurogenesis markers. Evidence strength: Limited = single or few independent primary studies; Mixed = condition- or context-dependent findings; Narrative synthesis = review-level integration without new primary data. Abbreviations: SVZ, subventricular zone; DG, dentate gyrus; PFC, prefrontal cortex; BrdU, bromodeoxyuridine; Ki67, proliferation marker; VBM, voxel-based morphometry; GD, gestation day; LD, lactation day.

**Table 3:** Hormonal and Neurochemical Modulators of Adult Hippocampal Neurogenesis.

Hormone/System	Species/Sex	Manipulation Context	Neurogenesis Endpoint (Region)	Direction of Effect	Evidence Type	Strength
Estradiol (17β-E2, E1, E3) [105]	Adult ovariectomized female rats	Acute administration (30 min–4 h exposure)	Cell proliferation (Dentate Gyrus)	↑ Proliferation (dose- and timing-dependent; transient)	Direct (BrdU)	Limited (acute paradigm)
Testosterone/DHT [86]	Adult male rats	Castration + androgen replacement (30 days)	Cell survival (Dentate Gyrus)	↑ Survival; no effect on proliferation	Direct (BrdU 30-day survival)	Consistent (within study)
Prolactin [87]	Adult female mice	Pregnancy-induced elevation; prolactin infusion	Proliferation (SVZ → OB)	↑ Proliferation (blocked by prolactin antagonist)	Direct (BrdU)	Limited (pregnancy context)
Oxytocin/Vasopressin [77]	Rodents (review)	Endogenous neuromodulation	No direct neurogenesis measures; synaptic modulation (hippocampus)	Modulate synaptic transmission	Indirect (review)	Indirect
BDNF/Neurotrophins [39]	Multiple species (review)	Activity-dependent expression	Supports survival & integration (DG)	↑ Survival/plasticity	Indirect–mechanistic	Review-based
Monoaminergic & Stress Pathways [48,49,104,118]	Rats/mice	Chronic social stress/isolation	Proliferation (mPFC; DG markers indirect)	↓ Proliferation (context-dependent)	Direct (BrdU in mPFC) + indirect hippocampal markers	Mixed/context-dependent

Evidence type: Direct = studies measuring adult neurogenesis markers (e.g., BrdU-based proliferation or survival assays); Indirect = synaptic, molecular, or circuit-level adaptations without direct neurogenesis endpoints; Review-based = narrative or integrative syntheses without primary cellular measurements. Direction of effect refers to reported changes relative to control or baseline conditions. Abbreviations: DG, dentate gyrus; SVZ, subventricular zone; mPFC, medial prefrontal cortex; DHT, dihydrotestosterone; BrdU, bromodeoxyuridine.

**Table 4:** Comparative species models linking social organization to adult neurogenesis.

Model/Species	Type of Social Organization	Direct Adult Neurogenesis Evidence (Region/Readout/Direction)	Social Context/Manipulation	Evidence Tier	References
Prairie vole (*Microtus ochrogaster*)	Social monogamy; stable pair bonds; biparental care	↑ Survival of adult-born cells (BrdU) in DG and olfactory bulb; sex-dependent effects	Cohabitation with mating; opposite-sex social exposure	Direct (experimental; sex-dependent)	[124,125]
Eusocial mole-rats (incl. naked mole-rat)	Eusocial colonies; reproductive castes	Low baseline hippocampal AHN (proliferation markers); slow maturation; status-related differences	Natural caste/status comparison (no acute manipulation)	Direct (comparative/ecological)	[126,127]
Nonhuman primates (macaques)	Complex hierarchies; long-term alliances	Presence of AHN in DG; markedly lower rates than rodents; prolonged maturation	No experimental social manipulation	Direct (descriptive; no social modulation tested)	[128,129]
Songbirds (oscine species)	Learned vocal communication; seasonal territoriality	Robust adult neurogenesis in HVC/RA; recruitment regulated by season and activity	Photoperiod; singing activity; social context	Direct (behavior-linked; strong evidence)	[130,131]
Teleost fish (e.g., zebrafish)	Shoaling/group living	Extensive adult proliferation across multiple zones; social valence modulates proliferation	Positive vs. negative social context; cortisol-independent	Direct (experimental social modulation)	[132,133]

Abbreviations: DG, dentate gyrus; HVC, high vocal center; RA, robust nucleus of the arcopallium; BrdU, 5-bromo-2′-deoxyuridine. Evidence tier: Direct = study measured adult neurogenesis markers. Comparative/ecological = interspecies comparison without experimental manipulation.

## Data Availability

No new data were created or analyzed in this study. Data sharing is not applicable to this article.

## Abbreviations

The following abbreviations are used in this manuscript:

AHN	Adult Hippocampal Neurogenesis
AVP	Arginine Vasopressin
BDNF	Brain-Derived Neurotrophic Factor
BrdU	Bromodeoxyuridine
CA1	Cornu Ammonis 1
CA2	Cornu Ammonis 2
CA3	Cornu Ammonis 3
DCX	Doublecortin
DG	Dentate Gyrus
dDG	Dorsal Dentate Gyrus
DHT	Dihydrotestosterone
EEG	Electroencephalography
ERα	Estrogen Receptor Alpha
ERβ	Estrogen Receptor Beta
HPA axis	Hypothalamic–Pituitary–Adrenal Axis
HPG axis	Hypothalamic–Pituitary–Gonadal Axis
HVC	High Vocal Center
LH	Luteinizing Hormone
mPFC	Medial Prefrontal Cortex
NAc	Nucleus Accumbens
NGF	Nerve Growth Factor
OXT	Oxytocin
PFC	Prefrontal Cortex
RA	Robust Nucleus of the Arcopallium
RNA-seq	RNA Sequencing
SVZ	Subventricular Zone
TrkB	Tropomyosin Receptor Kinase B
vDG	Ventral Dentate Gyrus
VTA	Ventral Tegmental Area
ΔFosB	Truncated FosB Transcription Factor
